# A quantitative study of stress fields ahead of a slip band blocked by a grain boundary in unalloyed magnesium

**DOI:** 10.1038/s41598-020-59684-y

**Published:** 2020-02-20

**Authors:** Mohsen Taheri Andani, Aaditya Lakshmanan, Mohammadreza Karamooz-Ravari, Veera Sundararaghavan, John Allison, Amit Misra

**Affiliations:** 1 Department of Mechanical Engineering, University of Michigan, Ann Arbor, MI 48105 USA; 2 Department of Materials Science and Engineering, University of Michigan, Ann Arbor, MI 48105 USA; 3 Department of Aerospace Engineering, University of Michigan, Ann Arbor, MI 48105 USA

**Keywords:** Mechanical properties, Metals and alloys

## Abstract

Stress localization ahead of a slip band blocked by a grain boundary is measured for three different grain boundaries in unalloyed Mg using high-resolution electron backscatter diffraction (HR-EBSD). The results are compared with a theoretical dislocation pile-up model, from which slip system resistance and micro-Hall–Petch coefficients for different grain boundary types are deduced. The results indicate that grain boundary character plays a crucial role in determining micro-Hall–Petch coefficients, which can be used to strengthen classical crystal plasticity constitutive models to make predictions linked to the effect of grain boundary strengthening.

## Introduction

Fundamental understanding of defect-defect interactions such as dislocations-grain boundaries (GBs)^1–3^, GBs-twins^4,5^, GBs-solute atoms^6–8^, dislocations-twins^9^, and dislocations-precipitates^10,11^ are key to assess the mechanical properties of polycrystalline materials. Among these interactions, grain boundaries play a crucial role in defining the strengthening of material^12–14^, fatigue crack initiation^15^, and stress corrosion cracking^16^. Under an applied load, dislocation glide accommodates plastic deformation until impeded by obstacles such as grain boundaries. The pileup of dislocations at a grain boundary successively increases the stress concentration until the boundary barrier to slip transmission is exceeded, resulting in slip transmission and further deformation^17^. Such a theory has been proposed to explain the empirical Hall-Petch equation^18–21^, which connects the yield strength of the bulk material to its average grain size: ${\sigma }_{y}={\sigma }_{0}+\frac{k}{\sqrt{L}}$ where ${\sigma }_{y}$ is the yield strength of the material, ${\sigma }_{0}$ is the friction stress, $L$ is the average grain size, and $k$ is the Hall-Petch coefficient which represents the grain boundary barrier to slip transmission. In connection with the microscopic phenomenon leading to the Hall-Petch effect, Weng^22^ proposed that the flow stress of a slip system may be expressed as $\tau ={\tau }^{\infty }+{k}_{\mu }.{d}^{-1/2}$, where at a specific strain, ${\tau }^{\infty }\,$represents the flow stress of a slip system in a free single crystal, $d$ is the grain size, and ${k}_{\mu }$ is some physical quantity that reflects the strength of the size-effect. This equation is referred to as the micro-Hall-Petch relationship in connection with the extension of the Hall-Petch equation to the slip system level in which the parameter $\,{k}_{\mu }$ is the micro-Hall-Petch coefficient^23,24^. Such a proposal is based on the physical argument by Armstrong *et al*.^25^ that in a polycrystalline material, dislocations approaching a grain boundary cannot freely cross the boundary and therefore a slip band can sustain higher stress compared to one in a single crystal. The role of individual grain boundary parameters (misorientation, tilt angle, twist angle, etc.) on slip band-GB interactions and its subsequent effect on the flow stress of a slip system and the strength of polycrystalline materials, has been theoretically studied^26,27^ but their calibration is primarily limited by the experimental technique to accurately predict the grain boundary energy or stress field induced by a blocked slip band at a grain boundary. Recently, high-resolution electron backscatter diffraction (HR-EBSD) developed by Wilkinson *et al*.^28^ enables the measurement of all components of the stress tensor in materials with spatial resolution on the order of 100 nm by assessing changes in Kikuchi diffraction patterns. Previous studies used this technique successfully to examine the residual stress concentration induced from a slip band blocked by a grain boundary in commercial purity titanium^29,30^ and irradiated steel^31,32^. In this work, HR-EBSD is used to compute the residual shear stress distribution ahead of slip band –grain boundary intersections in unalloyed Mg. The experimental results are used in conjunction with a continuum dislocation pile-up model from which the micro-Hall-Petch coefficients and slip system resistance for three different grain boundaries are reported. A simple phenomenological relationship to account for grain misorientation is proposed to foster the implementation of grain size effects on the critical resolved shear stresses used in crystal plasticity constitutive models. Obtaining such microstructural measurements will help to accurately calibrate the crystal plasticity finite element constitutive model to predict the mechanical response of magnesium alloys, considering both the grain size and geometrical features of grain boundaries.

## Results and Discussion

**Figure 1:**
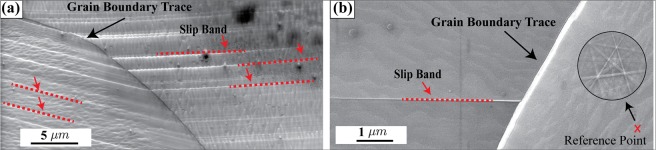
(**a**) SEM image of continuous slip band-grain boundary intersection. (**b**) SEM image of discontinuous slip band-grain boundary intersection. For generating the pileup-stress distribution ahead of grain boundary using CC4, a reference point toward the interior of the grain is selected. Red dashed lines denote slip band location.

The interaction of slip bands with grain boundaries is classified into two types: continuous slip bands (Fig. 1(a)) where slip is transmitted into the adjacent grain, and discontinuous slip bands (Fig. 1(b)) where the grain boundary blocks the slip bands^13^. The objective of this study is to calculate the shear stress distribution near the intersection of a blocked slip band with a grain boundary (i.e., a discontinuous slip band type interaction), and then fit it with the theoretical model to estimate the micro-Hall-Petch coefficient. The case of a continuous slip band that transmits from one grain to another unimpeded is not treated in this analysis. The cross-correlation algorithm described in the methods section is applied to calculate the stress states using the low-stress reference pattern marked with an X in Fig. 1(b). It must be noted that this reference point is in a state of low stress and not necessarily zero stress and Eq. 8 (see methods section) needs to be modified to take this into account. A closer look at Eq. 8 conveys that $\mathop{\mathrm{lim}}\limits_{X\to \infty }{\tau }_{p}(X)=0$ but the experimental data may not appear to asymptotically approach zero at sufficiently large $X$ simply because the reference point does not correspond to a zero-stress state. In other words, we need to account for a non-zero offset which can be accomplished by adding a constant term to the right-hand side of Eq. 8 which takes the form:

1 $${\tau }_{p}(X)=({\tau }_{0}^{\alpha }+\frac{{k}^{\alpha }}{\sqrt{L}})\,[\frac{X+\frac{L}{2}}{\sqrt{{(X+\frac{L}{2})}^{2}-{(\frac{L}{2})}^{2}}}-1]+{\rm{O}}$$

where O denotes the offset. This offset is assumed to not depend on $X$ and hence is a constant.

**Figure 2:**
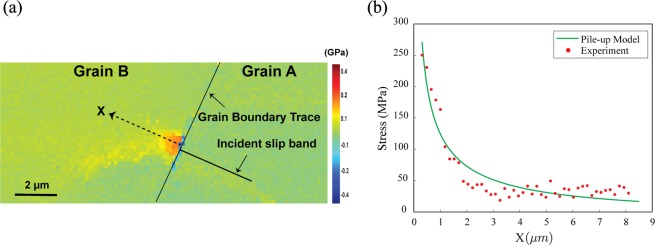
(**a**) Spatial variation of the residual shear stress resolved onto the active slip system of Grain A for a grain boundary with a misorientation angle of 62.8° (Boundary 1, grain size = 27 µm). The dashed line represents the direction along which the stress data is captured. (**b**) The residual shear stress profile ahead of a discontinuous dislocation-grain boundary intersection with comparison to the continuum dislocation pile-up model.

Figure 2(a) illustrates the stress concentration observed with HR-EBSD for a grain boundary with a misorientation of 62.8°. The slip system associated with the slip band is identified based on the maximum Schmid factor over all possible slip systems of Grain A, the orientation of which is available via EBSD. The analysis indicates the activation of the $(0\,0\,0\,1)[1\,\bar{2}\,1\,0]$ slip system in Grain A. A map of the residual shear stress field resolved onto the $(0\,0\,0\,1)[1\,\overline{2\,}\,1\,0]$ slip system of Grain A is shown in Fig. 2(a). A line scan direction along the slip band in Grain A extended into Grain B using the dashed line in Fig. 2(a) yields the residual shear stress profile in Grain B along the trace of the slip band. Resolving this stress along the slip system corresponding to the slip band in Grain A yields the point-plot (red points) in Fig. 2(b). The measured residual resolved shear stress is the highest close to the grain boundary and decreases as the distance from the grain boundary into the Grain B increases. This behavior was also observed by Guo *et al*.^30^ and Britton *et al*.^29^ in commercial purity titanium and by Johnson *et al*.^31–34^ for irradiated steels near blocked slip bands at grain boundaries. Knowing that basal slip is active, the value of ${\tau }_{0}^{\alpha }=0.5\,MPa$ is adopted from previous experimental studies on computing the yield stress for the basal slip system in Mg single crystals^35–38^. The experimental data is then fit with Eq. 7 using least squares. Accordingly, parameter values of ${s}^{\alpha }=73\pm 5\,{\rm{MPa}}$ and ${k}^{\alpha }=0.377\pm 0.04\,{\rm{MPa}}.{{\rm{m}}}^{1/2}$ are obtained.

**Figure 3:**
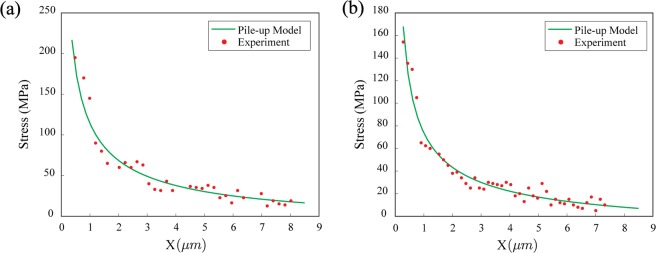
Residual shear stress ahead of blocked slip band fit with the continuum dislocation pile-up model for grain boundaries with misorientation angles of a) 41.3° (Boundary 2, grain size = 45 µm)and (**b**) 17.7° (Boundary 3, grain size = 85 µm).

The same procedure, as described for grain boundary shown in Fig. 2, is performed for two other boundaries, and the results are reported in Fig. 3. For comparison purposes, the fitting parameters and grain boundary misorientations for all three-grain boundaries are summarized in Table 1. By comparing the micro-Hall-Petch coefficients for three different grain boundaries, it is concluded that grain boundary character (tilt and twist angles) plays a significant role in defining this coefficient. It is also observed that the micro-Hall-Petch coefficient varies over a factor of two with increasing grain boundary misorientation: 17.7°, 41.3°, 62.8°. A comprehensive study with more grain boundary types is required to better understand the effect of grain boundary parameters on the micro-Hall-Petch coefficient. It is worth noting that the estimates of the micro-Hall-Petch coefficient obtained in this study, i.e., 0.209–0.377 MPa.m^1/2^, are in satisfactory agreement with previously reported values of the Hall-Petch coefficient for unalloyed Mg. For instance, Somekawa *et al*.^39^ obtained the value of 0.294 MPa.m^1/2^ from macroscopic compression tests on extruded unalloyed Mg at room temperature. Ono *et al*.^40^ studied the tensile deformation of rolled unalloyed Mg at 293 K and found a Hall-Petch coefficient of 0.291 MPa.m^1/2^.

**Table 1:** Comparison between the slip system resistance and micro-Hall-Petch coefficients of three different grain boundaries presented in Figs. 2 and 3.

	Boundary 1	Boundary 2	Boundary 3
Misorientation Angle(°)	62.8	41.3	17.7
${{\rm{s}}}^{{\rm{\alpha }}}$ (MPa)	73 ± 6	48 ± 5	23 ± 2
${{\rm{k}}}^{{\rm{\alpha }}}$ $({\rm{MPa}}.{{\rm{m}}}^{1/2}$)	0.377 ± 0.04	0.322 ± 0.03	0.209 ± 0.02
*ϕ* (°)	57.1	42.6	10.3
*k* (°)	31.4	1.9	14.6
*m*′	0.4632	0.7356	0.952

To describe the grain boundary dependence of the micro-Hall-Petch coefficient, we express ${k}^{\alpha }$ in terms of the geometric compatibility factor^41^, denoted by *m*′=cos(*ϕ*).cos(*k*), where $\phi$ and $\kappa$ represent the relative angle between the slip planes and slip directions, respectively, in the adjacent grains. In the present case, m′ is determined as follows. First, the slip system corresponding to the experimentally observed slip band in Grain A is identified. In the present study, for all three boundaries, the observed slip bands correspond to the $(0\,0\,0\,1)[1\,\bar{2}\,1\,0\,]$ slip system. Then the compatibility factors are estimated considering the $(0\,0\,0\,1)[1\,\bar{2}\,1\,0\,]$ in Grain A, and all the basal variants in Grain B. The maximum value among the three estimates is chosen as the value of *m*′ for that boundary as reported in Table 1. The effect of grain boundary misorientation on ${k}^{\alpha }$ can be accounted by assuming a phenomenological functional form. Such a form may be constructed based on the intuition that a lower geometric compatibility factor reflects lower slip system compatibility between grains, which enhances the contribution of the grain-boundary to the slip system resistance. A simple relationship used in the current analysis is the following

2 $${k}^{\alpha }={K}^{\alpha }{(1-m{\prime} )}^{c};c > 0$$

where ${K}^{\alpha }$ and $c$ are model parameters. The above expressions are then calibrated using data for misorientation angles 17.7° and 62.8° yielding ${K}^{\alpha }=0.4389$ and $c=0.2443$ after which the estimate of ${k}^{\alpha }$ for a misorientation angle of 41.3° equals 0.3171. This value is within 1.5% of the value determined in this study and provided in Table 1. Equation 2 can subsequently be used in conjunction with Eq. 8 in crystal plasticity models to incorporate phenomenologically, the effect of grain-size and misorientation on the initial slip-system resistance.

While the continuum dislocation pile-up model used here is quite simplistic, more realistic discrete models of double-ended dislocation pile-ups or continuum models for a finite number of double-ended pileups^42^ are not analytically tractable in general, leaving simulations as the only other alternative. There have been attempts to factor in the effect of grain boundaries based on dislocation-models of grain boundary ledges^43,44^, disclination models of high-angle grain boundaries^45^ and grain boundary energy-based models^46^. Future work will analyze the use of such models to improve our understanding of the HR-EBSD data. Such improvements, however, do not alter the fact that the micro-Hall Petch formula is empirical. A simple relationship as devised in Eq. 2 is suggested as a starting point for crystal plasticity calculations that account for the grain size effect.

## Conclusion

High-resolution electron backscatter diffraction technique, combined with a continuum dislocation pile-up model, is used to study the interaction of a slip band with grain boundary in unalloyed Mg. The proposed framework allows measurement of the slip system resistance of individual grains and determination of micro-Hall-Petch coefficients for different grain boundary types. The results indicate that the micro-Hall-Petch coefficient is sensitive to the grain boundary character. Accordingly, a simple phenomenological relationship is proposed relating the micro-Hall-Petch coefficient to the geometric compatibility factor. Such a relationship should be useful for implementing micro-Hall-Petch-based crystal plasticity constitutive models to predict grain boundary effects.

## Methods

### Materials and experimental methods

**Figure 4:**
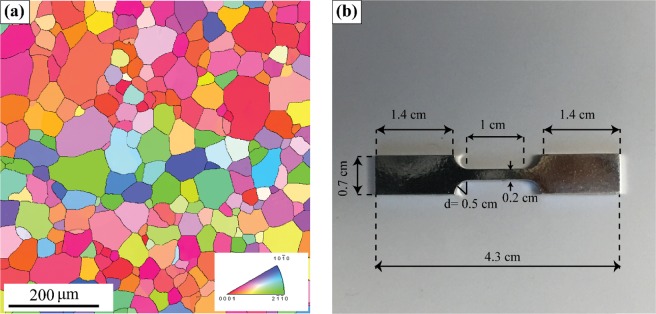
(**a**) EBSD orientation map and inverse pole figure of unalloyed Mg used in this study. The starting grain size is 45 $\pm 15\,\mu m$. (**b**) Tensile sample with a nominal thickness of 2 mm.

The material used in this study was extruded unalloyed Mg bar provided by Canmet Materials. The bar was extruded at 573 K to a diameter of 15 mm at the rate of 254 mm/min from an 85-mm diameter cast billet. The final average grain size is 45 $\pm 15\,\mu m$ as shown in Fig. 4(a). Tensile samples are machined following the design given in Fig. 4(b) and heat-treated at 250 °C for 24 h to minimize residual stress. The specimens are then polished using standard metallographic techniques via finishing with a 0.05 μm colloidal silica followed by etching with an acetic-nitric solution (5 mL nitric acid, 15 mL acetic acid, 20 mL water, and 60 mL ethanol). The polished samples are subjected to a tensile stress of 20 MPa. This relatively small stress amplitude has been selected to minimize the effect of work hardening so that we estimate the ‘initial’ slip system resistance as close as possible. The stress amplitude is also sufficiently low to preclude the occurrence of affects due to grain boundary sliding, which is typically observed at higher stresses or when the material is sufficiently deformed. After mechanical deformation, using a Tescan RISE scanning electron microscopy with the squared grid and 150 nm step size, high-resolution EBSD scans are performed near dislocation grain boundary interaction sites and Kikuchi patterns are collected. CrossCourt4 (CC4) software package developed by BLG Vantage is then used to calculate the full residual stress tensor following the cross-correlation algorithm developed by Britton and Wilkinson^47^. The stress tensor is then projected onto the active slip system to obtain the resolved shear stress on that slip system.

### Continuum dislocation pile-up model

**Figure 5:**
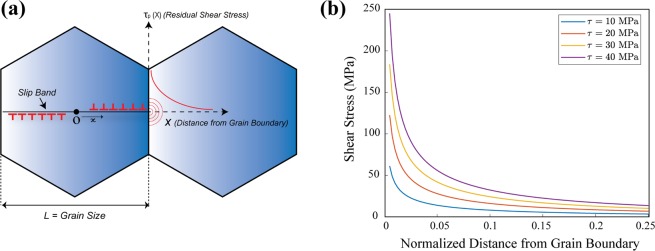
(**a**) Continuum model of dislocation pile-up at a grain boundary. The red curve represents the stress ahead of the pile-up based on Eq. 4. (**b**) Shear stress ahead of pile-up for different slip system resistance (${s}^{\alpha }$) - the pileup-stress increases proportionally with the resolved shear stress.

The following method to construct a model for a slip band based on the theory of continuous distribution of dislocations is adapted from Hirth and Lothe^48^. A slip band is idealized to a one-dimensional interval [−*L*/2, *L*/2], where the boundaries of the domain represent grain boundaries (Fig. 2(a)). At any point x ∈ [−*L*/2, *L*/2] a dislocation density field $\rho (x)\,$is prescribed, so that the total number of dislocations in a differential element $\delta x$ is $\delta n(x)=\rho (x)\delta x$. A continuous density field in one-dimension is a continuum representation of straight, infinite dislocations of positive and negative type, for a given Burgers vector. The sign of the density field refers to the group of dislocations of the corresponding sign. An applied stress field exerts a configurational force on the dislocation of the Peach- Koehler type. Additionally, there is a long-range stress field due to dislocations in the medium (by virtue of their presence), which imposes a configurational force on a dislocation present anywhere else in the medium. Any net force acting on the dislocations drives the system to an equilibrium state which is characterized by zero net configurational force. Because the expressions for the stress fields of dislocations are derived based on linear elasticity, the net configurational force is simply a sum of individual force terms. The equilibrium condition is then expressed mathematically as:

3 $$\tau (x)b+\frac{G{b}^{2}}{2\pi \kappa }{\int }_{-\frac{L}{2}}^{\frac{L}{2}}\frac{\rho (x{\prime} )}{x-x{\prime} }dx{\prime} =0$$

where $\tau (x)$ denotes the applied shear stress resolved along with the slip system, L is the grain size, G the shear modulus for an isotropic elastic material, b the Burgers vector strength, and $\kappa$ = 1 (for screw dislocations) or $\kappa \,$= 1 − $\upsilon$ (for edge dislocations). It is noted that unalloyed Mg is essentially elastically isotropic so that the Eq. 1 is a reasonable approximation. Now given $\,\tau (x)$, the density field $\rho (x)\,$satisfying the equilibrium equations needs to be computed. For this purpose, Eq. 1 is recast into a simpler mathematical form as follows

4 $$\begin{array}{c}\frac{1}{\pi }{\int }_{-1}^{1}\frac{f(\xi {\prime} )}{{\xi }^{\text{'}}-\xi }d\xi {\prime} =\frac{2\kappa \tau (\frac{L}{2}\xi )}{Gb}\\ \equiv { {\mathcal H} }_{\xi }[f(\xi {\prime} )]=\frac{2\kappa \tau (\frac{L}{2}\xi )}{Gb}\end{array}$$

where $=\,\frac{2x}{L}$, $\xi \text{'}=\frac{2x\text{'}}{L}$, $f(\xi {\prime} )=\rho (\frac{L}{2}\xi {\prime} )$ and ${ {\mathcal H} }_{\xi }[f(\xi {\prime} )]$ denotes the finite Hilbert transform of the function $f(\xi {\prime} )$ expressed in terms of the new variable $\xi$. Solving for the dislocation density field involves inverting the operator ${\mathcal H}$, a classical problem whose solution has been presented elsewhere^49,50^. A systematic procedure to invert the integral using some properties of Chebyshev polynomials when $\tau (x)\,$is a polynomial, is presented in the supplemental material. The special case of a spatially constant resolved shear stress $\tau (x)={\tau }_{0}$ is considered in this study resulting in

5 $$\begin{array}{c}{ {\mathcal H} }_{\xi }[f(\xi {\prime} )]=\frac{2\kappa {\tau }_{0}}{Gb}\\ \Rightarrow f(\xi {\prime} )=\frac{2\kappa {\tau }_{0}}{Gb}\frac{\xi {\prime} }{\sqrt{1-{\xi }^{\text{'}2}}}+\frac{C}{\sqrt{1-{\xi }^{\text{'}2}}}\\ \Rightarrow \rho (x)=\frac{2\kappa {\tau }_{0}}{Gb}\frac{x}{\sqrt{{(\frac{L}{2})}^{2}-{x}^{2}}}+\frac{CL}{2\sqrt{{(\frac{L}{2})}^{2}-{x}^{2}}}\end{array}$$

where C is a constant appearing due to the homogeneous solution of the integral equation. This constant can be related to the net Burgers vector (supplemental material) considering all the dislocations appearing in the slip band, which for simplicity is assumed to be zero. In other words, there is an equal number of dislocations of both positive and negative types. Accordingly, the stress ahead of the pile-up (pileup-stress) due to the dislocation distribution alone takes the form

6 $${\tau }_{p}(X)={\tau }_{0}[\frac{X+\frac{L}{2}}{\sqrt{{(X+\frac{L}{2})}^{2}-{(\frac{L}{2})}^{2}}}-1]$$

where $X=x-\frac{L}{2}$, is the distance from the grain boundary, as denoted in Fig. 5(a).

In comparison with experiments, it is noted that the theoretical prediction for the pileup-stress must not include the effect of the resolved stress, because the experiment measures the residual stress in the adjacent grain. This residual stress is considered to arise primarily from the development of a dislocation distribution in the slip band reminiscent of a dislocation pile-up. The sole purpose of the resolved shear stress is to generate this dislocation distribution which develops irreversibly, and hence, retains the functional form even after removal of the resolved shear stress. In other words, the generated dislocation distribution is assumed to change negligibly so that the form of the pileup stress is not affected significantly. These simplifications are debatable but in the interest of obtaining a simple analytical form, are suggested to be an appropriate starting point.

The function ${\tau }_{p}(X)$ (stress ahead of the pile-up) for different values of ${\tau }_{0}$ is plotted in Fig. 5(b).

To purport a particular form of the resolved shear stress, two assumptions are made.

1. The resolved shear stress on the slip system equals the initial slip system resistance, which arises by neglecting the phenomenon of work hardening on that slip system. In other words, the applied shear stress required to equilibrate a dislocation distribution is identical to the initial slip system resistance which must be overcome to produce the slip band and accommodating the majority of the applied deformation.
2. It is assumed that classical Hall-Petch relationship may be extended to the slip system level, formerly termed as “micro-Hall-Petch” relation^22,25^. It is one way of separating the contribution of grain size from the local lattice resistance, in the initial slip system resistance. Additionally, because the pile-up model doesn’t take into account the grain boundary character, the grain boundary effect is subsumed in the estimates of the micro-Hall-Petch coefficients. Accordingly, the slip system resistance is expressed in the following form:

7 $${s}^{\alpha }={\tau }_{0}^{\alpha }+\frac{{k}^{\alpha }}{\sqrt{L}}$$

where ${\tau }_{0}^{\alpha }$ is the flow stress of slip system $\alpha$ of a theoretically infinite single crystal, ${k}^{\alpha }$ the micro-Hall-Petch coefficient of the slip system $\alpha$ signifying the strength of the size effect and $L$ is the slip system-level grain size, which in this case represents the length of the slip band across an entire grain. In the context of the current experiment, $L$ corresponds to the length of the slip trace measured along the direction perpendicular to the dislocation(infinite edge or screw) line and slip plane normal of the slip system from one-grain boundary to the opposite. Subsequently, we refer to $\,L$ as the grain size. Then Substituting Eq. 5 in Eq. 4 yields:

8 $${\tau }_{p}(X)=({\tau }_{0}^{\alpha }+\frac{{k}^{\alpha }}{\sqrt{L}})[\frac{X+\frac{L}{2}}{\sqrt{{(X+\frac{L}{2})}^{2}-{(\frac{L}{2})}^{2}}}-1]$$

## Supplementary information

Supplementary Data.
