# Supplementary material for: A quantitative study of stress fields ahead of a slip band blocked by a grain boundary in unalloyed magnesium
Source: Sci Rep. 2020 Feb 20;10:3084. doi: 10.1038/s41598-020-59684-y (PMC7033223; doi:10.1038/s41598-020-59684-y)
Supplement: Supplementary file 1 — Supplementary Data. [file 41598_2020_59684_MOESM1_ESM.docx]

SUPPLEMENTARY INFORMATION

A quantitative study of stress fields ahead of a slip band blocked by a grain boundary in unalloyed magnesium

Authors: Mohsen Taheri Andani^a,b,*^, Aaditya Lakshmanan^c^, Mohammadreza Karamooz-Ravari^a^, Veera Sundararaghavan^b,c^, John Allison^b^, Amit Misra^a,b^

^a^ Department of Mechanical Engineering, University of Michigan, Ann Arbor, MI, 48105, USA

^b^ Department of Materials Science and Engineering, University of Michigan, Ann Arbor, MI, 48105, USA

^c^ Department of Aerospace Engineering, University of Michigan, Ann Arbor, MI, 48105, USA

*Correspondence and requests for materials should be addressed to M.T.A (email: mtaheri@umich.edu)

The Chebyshev polynomials of the 1st and 2nd kind are defined as follows:

Polynomial of the 1st kind: $T_{n}\left( \cos\theta\right)=\cos\left( n\theta\right);n \geq0 , n \in Z$

Polynomial of the 2nd kind: $U_{n}\left( \cos\theta\right)=\frac{sin((n+1)\theta)}{sin(\theta)} ;n \geq-1 , n \in Z$

To demonstrate how these polynomials are obtained, a couple of examples are considered. Let’s say we wish to compute $T_{3}(x)$ and $U_{2}(x)$. Then using the trigonometric identities

$$T_{3}\left( \cos(\theta) \right)=\cos\left( 3\theta\right)$$

$$=4 \cos^{3} (\theta)-3 \cos(\theta)$$

$$\Longrightarrow T_{3}\left( x \right)=4 x^{3}-3 x$$

$$U_{2}\left( \cos\theta\right)=\frac{\sin\left( 3\theta\right)}{\sin\left( \theta\right)}$$

$$=\frac{3\sin\left( \theta\right)-4 \sin^{3}(\theta))}{\sin\left( \theta\right)}$$

$$=3-4 \sin^{2}\left( \theta\right)$$

$$=4 \cos^{2} \left( \theta\right)-1$$

$$\Longrightarrow U_{2}\left( x \right)=4 x^{2}-1$$

It is important to note that the Chebyshev polynomials form a complete basis of the set of polynomials. In other words, any polynomial can be expressed uniquely as a linear combination of Chebyshev polynomials (of either kind). An additional property that will be used to set up the procedure is the following:

$\mathcal{H}_{\xi}\left[ \frac{T_{n}(\xi^{'})}{\sqrt{1-\xi^{'2}}} \right]=U_{n-1}(\xi)$ (s1)

We note specifically that $U_{-1}\left( \xi\right)=0$ so that $\mathcal{H}_{\xi}\left[ \frac{T_{1}(\xi^{'})}{\sqrt{1-\xi^{'2}}} \right]=\mathcal{H}_{\xi}\left[ \frac{1}{\sqrt{1-\xi^{'2}}} \right]=U_{-1}\left( \xi\right)=0$. This relation contributes to the homogeneous part of the solution appearing in Eqn.s3. For the special case that $\tau(x)$ is a polynomial, the procedure to invert the integral exactly is described as follows:

1. Start with the RHS of Eqn.s2 and let $g\left( \xi\right):=\tau\left( \frac{L}{2}\xi\right)$. Because $\tau\left( \frac{L}{2}\xi\right)$ is a polynomial, so is $g\left( \xi\right)$. Due to the completeness of the Chebyshev polynomials of the 2nd kind, express $g\left( \xi\right)$ as a linear combination of them. Then we have

$$g\left( \xi\right)=\sum_{i=0}^{k} a_{k}U_{k}\left( \xi\right)$$

2. Using Eqn.s1 we have

$$g\left( \xi\right)=\sum_{i=0}^{k} a_{i}U_{i}\left( \xi\right)$$

$=\sum_{i=0}^{k} a_{i}\mathcal{H}_{\xi}\left[ \frac{T_{i+1}\left( \xi^{'} \right)}{\sqrt{1-\xi^{'2}}} \right]+c\mathcal{H}_{\xi}\left[ \frac{1}{\sqrt{1-\xi^{'2}}} \right]$

$$=\mathcal{H}_{\xi}\left[ \sum_{i=0}^{k} a_{i}\frac{T_{i+1}\left( \xi^{'} \right)}{\sqrt{1-\xi^{'2}}}+\frac{c}{\sqrt{1-\xi^{'2}}} \right]$$

$$\Longrightarrow\frac{2\kappa}{Gb}g\left( \xi\right)=\mathcal{H}_{\xi}\left[ \sum_{i=0}^{k} \frac{2\kappa a_{i}}{Gb}\frac{T_{i+1}\left( \xi^{'} \right)}{\sqrt{1-\xi^{'2}}}+\frac{2\kappa c}{Gb\sqrt{1-\xi^{'2}}} \right]$$

$$\Longrightarrow\mathcal{H}_{\xi}\left[ f\left( \xi^{'} \right) \right]=\mathcal{H}_{\xi}\left[ \sum_{i=0}^{k} \frac{2\kappa a_{i}}{Gb}\frac{T_{i+1}\left( \xi^{'} \right)}{\sqrt{1-\xi^{'2}}}+\frac{2\kappa c}{Gb\sqrt{1-\xi^{'2}}} \right]$$

$$\Longrightarrow f\left( \xi^{'} \right)=\sum_{i=0}^{k} \frac{2\kappa a_{i}}{Gb}\frac{T_{i+1}\left( \xi^{'} \right)}{\sqrt{1-\xi^{'2}}}+\frac{2\kappa c}{Gb\sqrt{1-\xi^{'2}}}$$

1. The equilibrium dislocation density can then be expressed as

$$\rho\left( x \right)=f\left( \frac{x}{L} \right)=\sum_{i=0}^{k} \frac{2\kappa a_{i}}{Gb}\frac{T_{i+1}\left( \frac{2x}{L} \right)}{\sqrt{\left( \frac{L}{2} \right)^{2}-x^{2}}}+\frac{\kappa Lc}{Gb\sqrt{\left( \frac{L}{2} \right)^{2}-x^{2}}}$$

2. The stress field ahead of the pile-up due to the dislocation density alone then turns out as follows

$$\tau_{p}\left( x \right)=\sum_{i=0}^{k} a_{i}\frac{T_{i+1}\left( \frac{2x}{L} \right)}{\sqrt{\left( \frac{L}{2} \right)^{2}-x^{2}}}- \tau(x)$$

The above procedure is implemented in a MATLAB script using Chebfun [1], an open-source software system for numerical computing with functions.

1. Driscoll TA, Hale N, Trefethen LN. Chebfun guide. Pafnuty Publications, Oxford; 2014.
